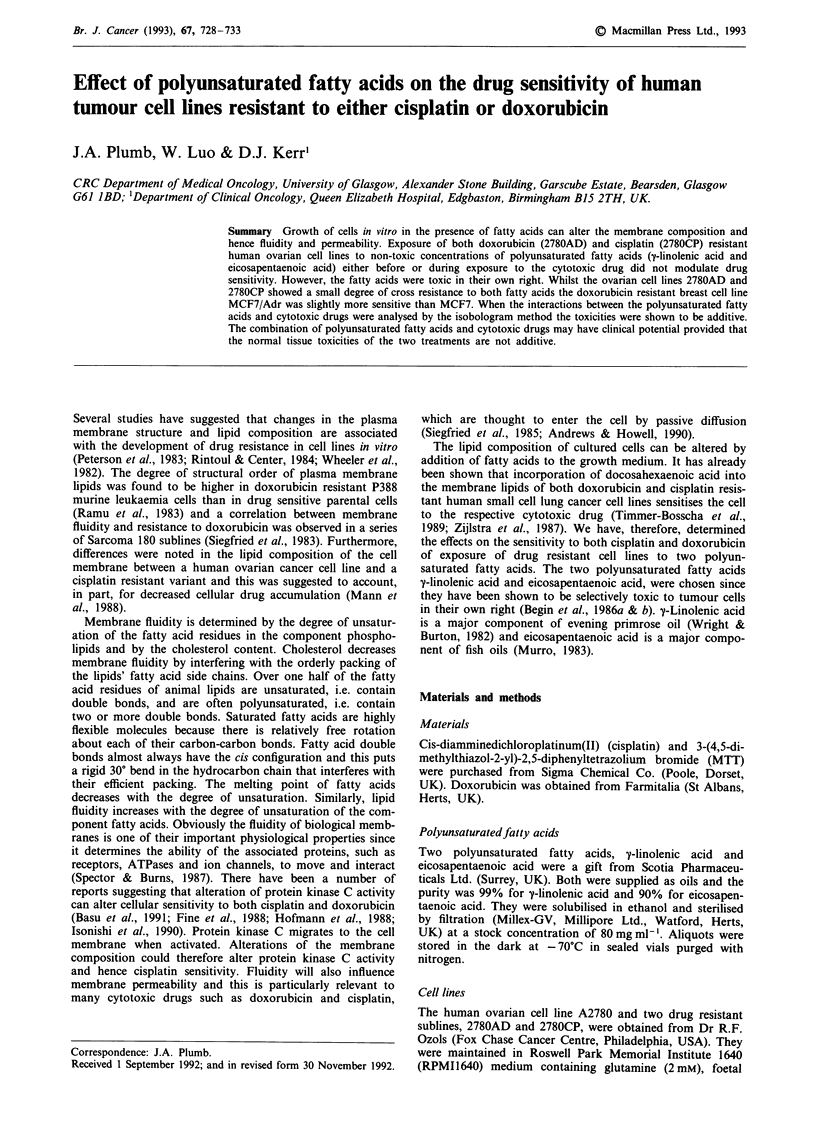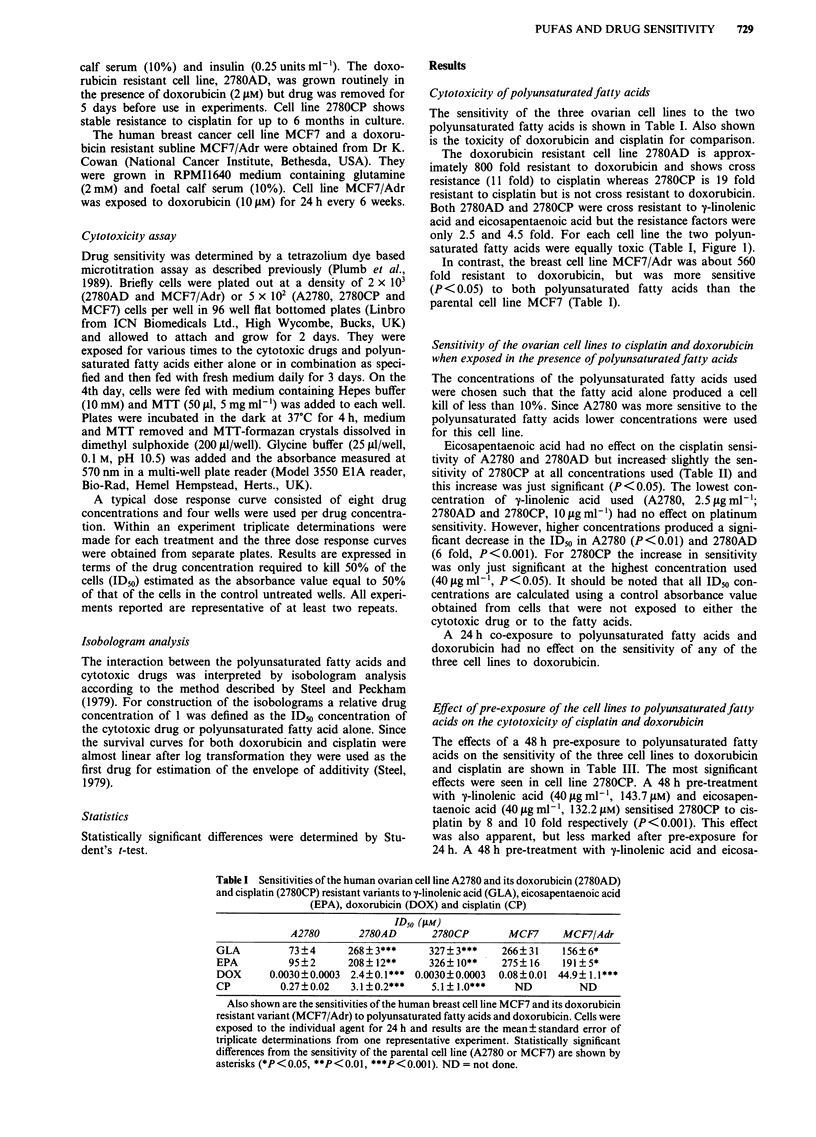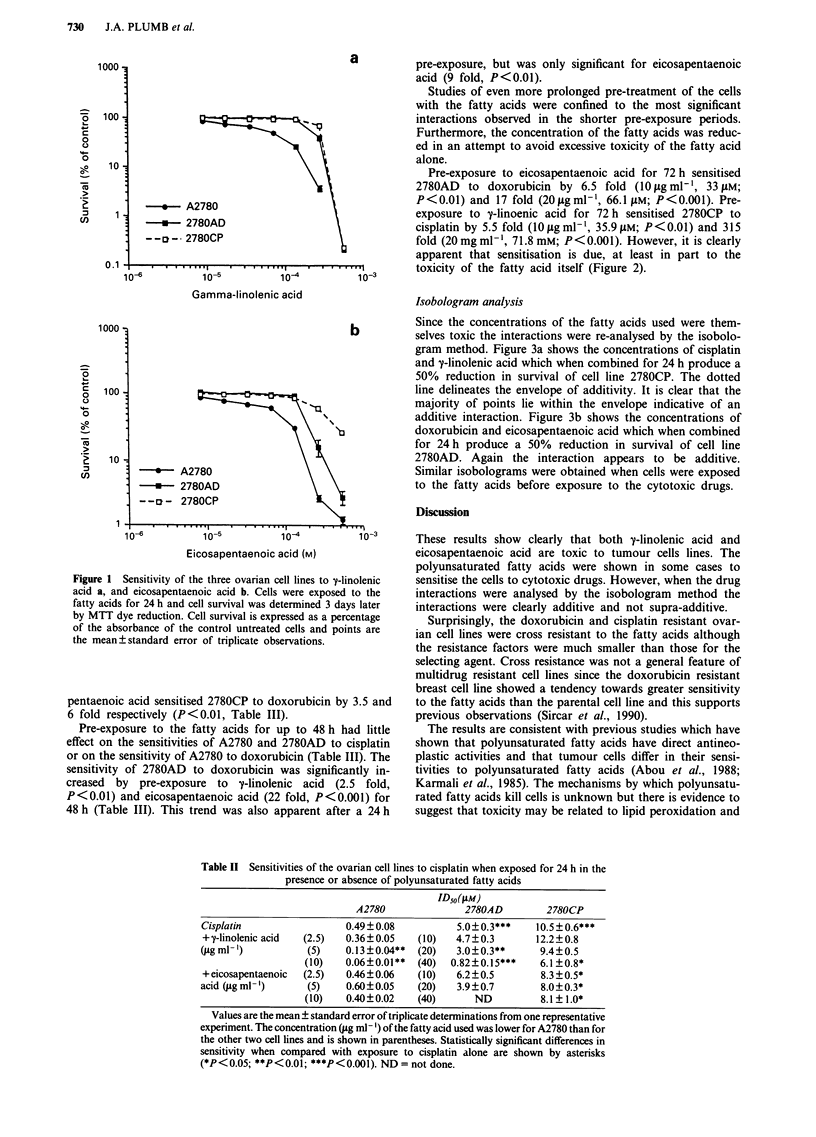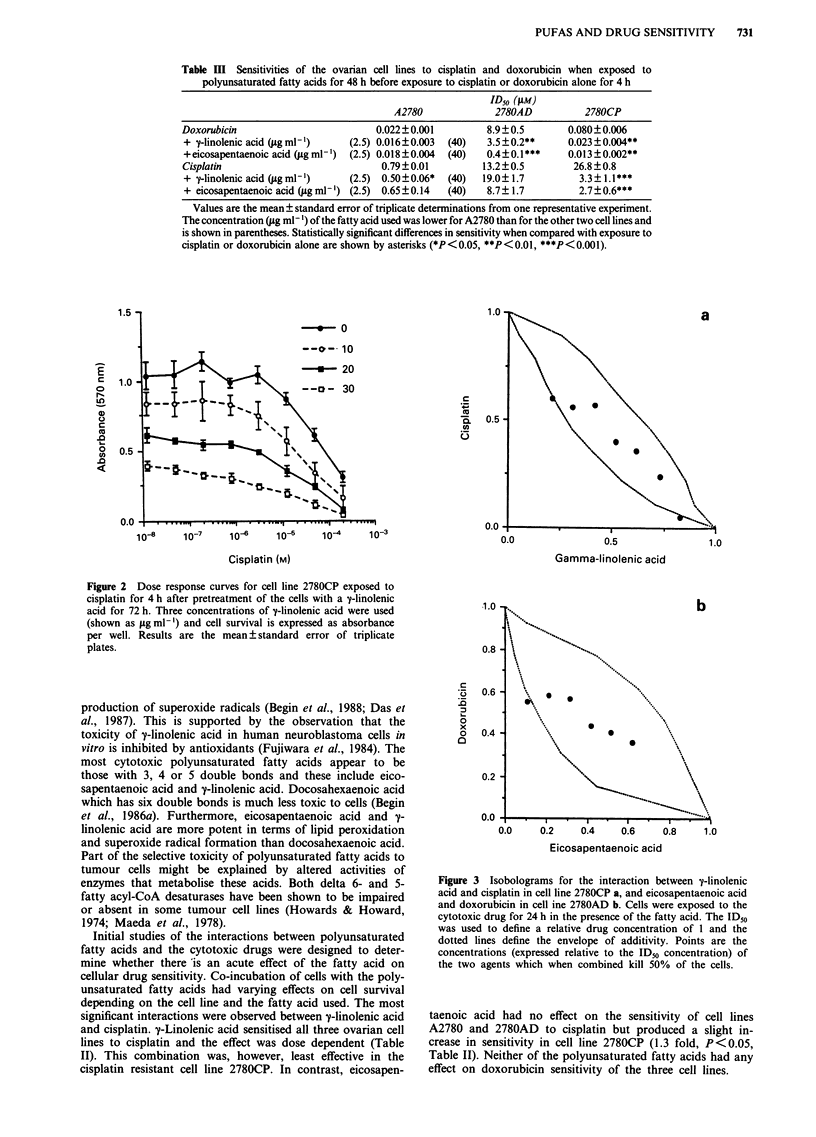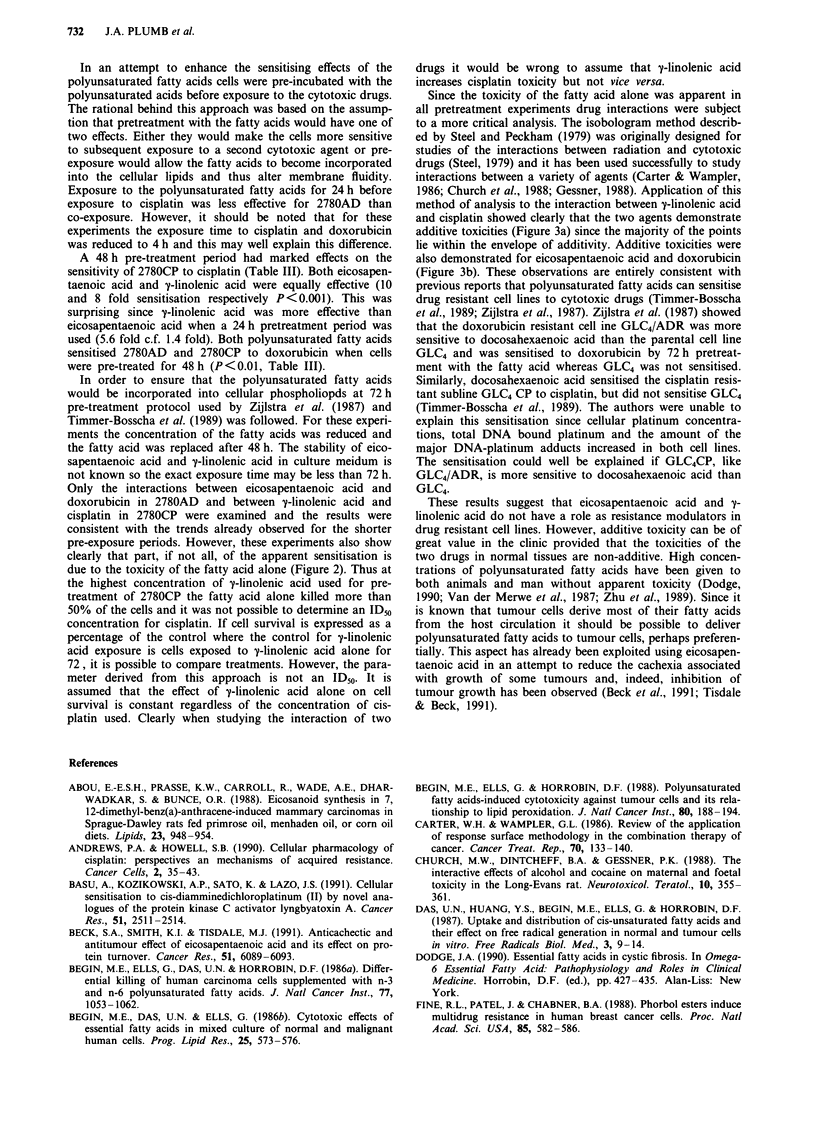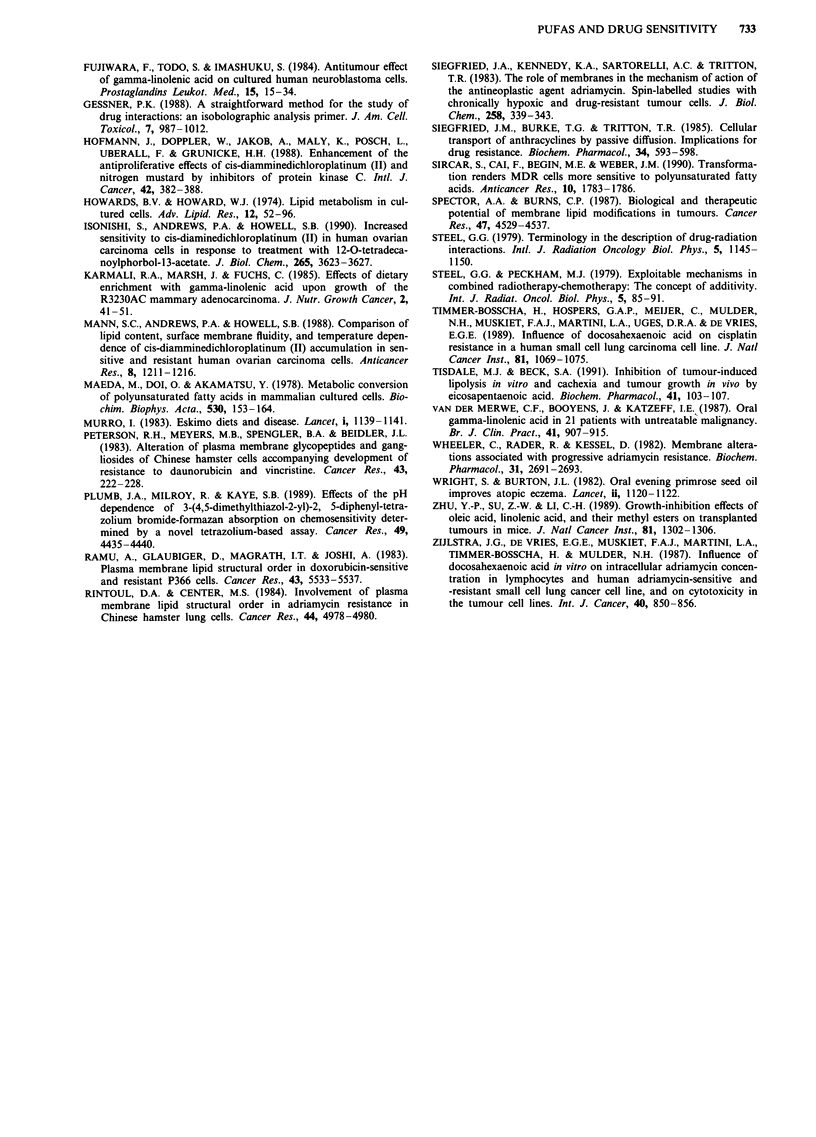# Effect of polyunsaturated fatty acids on the drug sensitivity of human tumour cell lines resistant to either cisplatin or doxorubicin.

**DOI:** 10.1038/bjc.1993.133

**Published:** 1993-04

**Authors:** J. A. Plumb, W. Luo, D. J. Kerr

**Affiliations:** CRC Department of Medical Oncology, University of Glasgow, Bearsden, UK.

## Abstract

Growth of cells in vitro in the presence of fatty acids can alter the membrane composition and hence fluidity and permeability. Exposure of both doxorubicin (2780AD) and cisplatin (2780CP) resistant human ovarian cell lines to non-toxic concentrations of polyunsaturated fatty acids (gamma-linolenic acid and eicosapentaenoic acid) either before or during exposure to the cytotoxic drug did not modulate drug sensitivity. However, the fatty acids were toxic in their own right. Whilst the ovarian cell lines 2780AD and 2780CP showed a small degree of cross resistance to both fatty acids the doxorubicin resistant breast cell line MCF7/Adr was slightly more sensitive than MCF7. When the interactions between the polyunsaturated fatty acids and cytotoxic drugs were analysed by the isobologram method the toxicities were shown to be additive. The combination of polyunsaturated fatty acids and cytotoxic drugs may have clinical potential provided that the normal tissue toxicities of the two treatments are not additive.


Br. J. Cancer (1993), 67, 728–733 © Macmillan Press Ltd., 1993

### Effect of polyunsaturated fatty acids on the drug sensitivity of human tumour cell lines resistant to either cisplatin or doxorubicin

J.A. Plumb, W. Luo & D.J. Kerr[1]

CRC Department of Medical Oncology, University of Glasgow, Alexander Stone Building, Garscube Estate, Bearsden, Glasgow G61 1BD; [1]Department of Clinical Oncology, Queen Elizabeth Hospital, Edgbaston, Birmingham B15 2TH, UK.

**Summary** Growth of cells *in vitro* in the presence of fatty acids can alter the membrane composition and hence fluidity and permeability. Exposure of both doxorubicin (2780AD) and cisplatin (2780CP) resistant human ovarian cell lines to non-toxic concentrations of polyunsaturated fatty acids (γ-linolenic acid and eicosapentaenoic acid) either before or during exposure to the cytotoxic drug did not modulate drug sensitivity. However, the fatty acids were toxic in their own right. Whilst the ovarian cell lines 2780AD and 2780CP showed a small degree of cross resistance to both fatty acids the doxorubicin resistant breast cell line MCF7/Adr was slightly more sensitive than MCF7. When the interactions between the polyunsaturated fatty acids and cytotoxic drugs were analysed by the isobologram method the toxicities were shown to be additive. The combination of polyunsaturated fatty acids and cytotoxic drugs may have clinical potential provided that the normal tissue toxicities of the two treatments are not additive.

Several studies have suggested that changes in the plasma membrane structure and lipid composition are associated with the development of drug resistance in cell lines *in vitro* (Peterson *et al.*, 1983; Rintoul & Center, 1984; Wheeler *et al.*, 1982). The degree of structural order of plasma membrane lipids was found to be higher in doxorubicin resistant P388 murine leukaemia cells than in drug sensitive parental cells (Ramu *et al.*, 1983) and a correlation between membrane fluidity and resistance to doxorubicin was observed in a series of Sarcoma 180 sublines (Siegfried *et al.*, 1983). Furthermore, differences were noted in the lipid composition of the cell membrane between a human ovarian cancer cell line and a cisplatin resistant variant and this was suggested to account, in part, for decreased cellular drug accumulation (Mann *et al.*, 1988).

Membrane fluidity is determined by the degree of unsaturation of the fatty acid residues in the component phospholipids and by the cholesterol content. Cholesterol decreases membrane fluidity by interfering with the orderly packing of the lipids' fatty acid side chains. Over one half of the fatty acid residues of animal lipids are unsaturated, i.e. contain double bonds, and are often polyunsaturated, i.e. contain two or more double bonds. Saturated fatty acids are highly flexible molecules because there is relatively free rotation about each of their carbon-carbon bonds. Fatty acid double bonds almost always have the *cis* configuration and this puts a rigid 30° bend in the hydrocarbon chain that interferes with their efficient packing. The melting point of fatty acids decreases with the degree of unsaturation. Similarly, lipid fluidity increases with the degree of unsaturation of the component fatty acids. Obviously the fluidity of biological membranes is one of their important physiological properties since it determines the ability of the associated proteins, such as receptors, ATPases and ion channels, to move and interact (Spector & Burns, 1987). There have been a number of reports suggesting that alteration of protein kinase C activity can alter cellular sensitivity to both cisplatin and doxorubicin (Basu *et al.*, 1991; Fine *et al.*, 1988; Hofmann *et al.*, 1988; Isonishi *et al.*, 1990). Protein kinase C migrates to the cell membrane when activated. Alterations of the membrane composition could therefore alter protein kinase C activity and hence cisplatin sensitivity. Fluidity will also influence membrane permeability and this is particularly relevant to many cytotoxic drugs such as doxorubicin and cisplatin, which are thought to enter the cell by passive diffusion (Siegfried *et al.*, 1985; Andrews & Howell, 1990).

The lipid composition of cultured cells can be altered by addition of fatty acids to the growth medium. It has already been shown that incorporation of docosahexaenoic acid into the membrane lipids of both doxorubicin and cisplatin resistant human small cell lung cancer cell lines sensitises the cell to the respective cytotoxic drug (Timmer-Bosscha *et al.*, 1989; Zijlstra *et al.*, 1987). We have, therefore, determined the effects on the sensitivity to both cisplatin and doxorubicin of exposure of drug resistant cell lines to two polyunsaturated fatty acids. The two polyunsaturated fatty acids γ-linolenic acid and eicosapentaenoic acid, were chosen since they have been shown to be selectively toxic to tumour cells in their own right (Begin *et al.*, 1986*a* & *b*). γ-Linolenic acid is a major component of evening primrose oil (Wright & Burton, 1982) and eicosapentaenoic acid is a major component of fish oils (Murro, 1983).

### Materials and methods

### Materials

Cis-diamminedichloroplatinum(II) (cisplatin) and 3-(4,5-dimethylthiazol-2-yl)-2,5-diphenyltetrazolium bromide (MTT) were purchased from Sigma Chemical Co. (Poole, Dorset, UK). Doxorubicin was obtained from Farmitalia (St Albans, Herts, UK).

### Polyunsaturated fatty acids

Two polyunsaturated fatty acids, γ-linolenic acid and eicosapentaenoic acid were a gift from Scotia Pharmaceuticals Ltd. (Surrey, UK). Both were supplied as oils and the purity was 99% for γ-linolenic acid and 90% for eicosapentaenoic acid. They were solubilised in ethanol and sterilised by filtration (Millex-GV, Millipore Ltd., Watford, Herts, UK) at a stock concentration of 80 mg ml$^{-1}$. Aliquots were stored in the dark at −70°C in sealed vials purged with nitrogen.

### Cell lines

The human ovarian cell line A2780 and two drug resistant sublines, 2780AD and 2780CP, were obtained from Dr R.F. Ozols (Fox Chase Cancer Centre, Philadelphia, USA). They were maintained in Roswell Park Memorial Institute 1640 (RPMI1640) medium containing glutamine (2 mM), foetal

Correspondence: J.A. Plumb.
Received 1 September 1992; and in revised form 30 November 1992.

calf serum (10%) and insulin (0.25 units $ml^{-1}$). The doxorubicin resistant cell line, 2780AD, was grown routinely in the presence of doxorubicin (2 μM) but drug was removed for 5 days before use in experiments. Cell line 2780CP shows stable resistance to cisplatin for up to 6 months in culture.

The human breast cancer cell line MCF7 and a doxorubicin resistant subline MCF7/Adr were obtained from Dr K. Cowan (National Cancer Institute, Bethesda, USA). They were grown in RPMI1640 medium containing glutamine (2 mM) and foetal calf serum (10%). Cell line MCF7/Adr was exposed to doxorubicin (10 μM) for 24 h every 6 weeks.

### *Cytotoxicity assay*

Drug sensitivity was determined by a tetrazolium dye based microtitration assay as described previously (Plumb *et al.*, 1989). Briefly cells were plated out at a density of $2 \times 10^3$ (2780AD and MCF7/Adr) or $5 \times 10^2$ (A2780, 2780CP and MCF7) cells per well in 96 well flat bottomed plates (Linbro from ICN Biomedicals Ltd., High Wycombe, Bucks, UK) and allowed to attach and grow for 2 days. They were exposed for various times to the cytotoxic drugs and polyunsaturated fatty acids either alone or in combination as specified and then fed with fresh medium daily for 3 days. On the 4th day, cells were fed with medium containing Hepes buffer (10 mM) and MTT (50 μl, 5 mg $ml^{-1}$) was added to each well. Plates were incubated in the dark at 37°C for 4 h, medium and MTT removed and MTT-formazan crystals dissolved in dimethyl sulphoxide (200 μl/well). Glycine buffer (25 μl/well, 0.1 M, pH 10.5) was added and the absorbance measured at 570 nm in a multi-well plate reader (Model 3550 E1A reader, Bio-Rad, Hemel Hempstead, Herts., UK).

A typical dose response curve consisted of eight drug concentrations and four wells were used per drug concentration. Within an experiment triplicate determinations were made for each treatment and the three dose response curves were obtained from separate plates. Results are expressed in terms of the drug concentration required to kill 50% of the cells ($ID_{50}$) estimated as the absorbance value equal to 50% of that of the cells in the control untreated wells. All experiments reported are representative of at least two repeats.

### *Isobologram analysis*

The interaction between the polyunsaturated fatty acids and cytotoxic drugs was interpreted by isobologram analysis according to the method described by Steel and Peckham (1979). For construction of the isobolograms a relative drug concentration of 1 was defined as the $ID_{50}$ concentration of the cytotoxic drug or polyunsaturated fatty acid alone. Since the survival curves for both doxorubicin and cisplatin were almost linear after log transformation they were used as the first drug for estimation of the envelope of additivity (Steel, 1979).

### *Statistics*

Statistically significant differences were determined by Student's *t*-test.

### Results

### *Cytotoxicity of polyunsaturated fatty acids*

The sensitivity of the three ovarian cell lines to the two polyunsaturated fatty acids is shown in Table I. Also shown is the toxicity of doxorubicin and cisplatin for comparison.

The doxorubicin resistant cell line 2780AD is approximately 800 fold resistant to doxorubicin and shows cross resistance (11 fold) to cisplatin whereas 2780CP is 19 fold resistant to cisplatin but is not cross resistant to doxorubicin. Both 2780AD and 2780CP were cross resistant to γ-linolenic acid and eicosapentaenoic acid but the resistance factors were only 2.5 and 4.5 fold. For each cell line the two polyunsaturated fatty acids were equally toxic (Table I, Figure 1).

In contrast, the breast cell line MCF7/Adr was about 560 fold resistant to doxorubicin, but was more sensitive ($P<0.05$) to both polyunsaturated fatty acids than the parental cell line MCF7 (Table I).

### *Sensitivity of the ovarian cell lines to cisplatin and doxorubicin when exposed in the presence of polyunsaturated fatty acids*

The concentrations of the polyunsaturated fatty acids used were chosen such that the fatty acid alone produced a cell kill of less than 10%. Since A2780 was more sensitive to the polyunsaturated fatty acids lower concentrations were used for this cell line.

Eicosapentaenoic acid had no effect on the cisplatin sensitivity of A2780 and 2780AD but increased slightly the sensitivity of 2780CP at all concentrations used (Table II) and this increase was just significant ($P<0.05$). The lowest concentration of γ-linolenic acid used (A2780, 2.5 μg $ml^{-1}$; 2780AD and 2780CP, 10 μg $ml^{-1}$) had no effect on platinum sensitivity. However, higher concentrations produced a significant decrease in the $ID_{50}$ in A2780 ($P<0.01$) and 2780AD (6 fold, $P<0.001$). For 2780CP the increase in sensitivity was only just significant at the highest concentration used (40 μg $ml^{-1}$, $P<0.05$). It should be noted that all $ID_{50}$ concentrations are calculated using a control absorbance value obtained from cells that were not exposed to either the cytotoxic drug or to the fatty acids.

A 24 h co-exposure to polyunsaturated fatty acids and doxorubicin had no effect on the sensitivity of any of the three cell lines to doxorubicin.

### *Effect of pre-exposure of the cell lines to polyunsaturated fatty acids on the cytotoxicity of cisplatin and doxorubicin*

The effects of a 48 h pre-exposure to polyunsaturated fatty acids on the sensitivity of the three cell lines to doxorubicin and cisplatin are shown in Table III. The most significant effects were seen in cell line 2780CP. A 48 h pre-treatment with γ-linolenic acid (40 μg $ml^{-1}$, 143.7 μM) and eicosapentaenoic acid (40 μg $ml^{-1}$, 132.2 μM) sensitised 2780CP to cisplatin by 8 and 10 fold respectively ($P<0.001$). This effect was also apparent, but less marked after pre-exposure for 24 h. A 48 h pre-treatment with γ-linolenic acid and eicosa-

**Table I** Sensitivities of the human ovarian cell line A2780 and its doxorubicin (2780AD) and cisplatin (2780CP) resistant variants to γ-linolenic acid (GLA), eicosapentaenoic acid (EPA), doxorubicin (DOX) and cisplatin (CP)

| | $ID_{50}$ (μM) | | | | |
|---|---|---|---|---|---|
| | *A2780* | *2780AD* | *2780CP* | *MCF7* | *MCF7/Adr* |
| GLA | 73±4 | 268±3*** | 327±3*** | 266±31 | 156±6* |
| EPA | 95±2 | 208±12** | 326±10** | 275±16 | 191±5* |
| DOX | 0.0030±0.0003 | 2.4±0.1*** | 0.0030±0.0003 | 0.08±0.01 | 44.9±1.1*** |
| CP | 0.27±0.02 | 3.1±0.2*** | 5.1±1.0*** | ND | ND |

Also shown are the sensitivities of the human breast cell line MCF7 and its doxorubicin resistant variant (MCF7/Adr) to polyunsaturated fatty acids and doxorubicin. Cells were exposed to the individual agent for 24 h and results are the mean±standard error of triplicate determinations from one representative experiment. Statistically significant differences from the sensitivity of the parental cell line (A2780 or MCF7) are shown by asterisks (*$P<0.05$, **$P<0.01$, ***$P<0.001$). ND = not done.

**Figure 1** Sensitivity of the three ovarian cell lines to γ-linolenic acid **a**, and eicosapentaenoic acid **b**. Cells were exposed to the fatty acids for 24 h and cell survival was determined 3 days later by MTT dye reduction. Cell survival is expressed as a percentage of the absorbance of the control untreated cells and points are the mean ± standard error of triplicate observations.

pentaenoic acid sensitised 2780CP to doxorubicin by 3.5 and 6 fold respectively ($P<0.01$, Table III).

Pre-exposure to the fatty acids for up to 48 h had little effect on the sensitivities of A2780 and 2780AD to cisplatin or on the sensitivity of A2780 to doxorubicin (Table III). The sensitivity of 2780AD to doxorubicin was significantly increased by pre-exposure to γ-linolenic acid (2.5 fold, $P<0.01$) and eicosapentaenoic acid (22 fold, $P<0.001$) for 48 h (Table III). This trend was also apparent after a 24 h pre-exposure, but was only significant for eicosapentaenoic acid (9 fold, $P<0.01$).

Studies of even more prolonged pre-treatment of the cells with the fatty acids were confined to the most significant interactions observed in the shorter pre-exposure periods. Furthermore, the concentration of the fatty acids was reduced in an attempt to avoid excessive toxicity of the fatty acid alone.

Pre-exposure to eicosapentaenoic acid for 72 h sensitised 2780AD to doxorubicin by 6.5 fold (10 μg ml$^{-1}$, 33 μM; $P<0.01$) and 17 fold (20 μg ml$^{-1}$, 66.1 μM; $P<0.001$). Pre-exposure to γ-linoenic acid for 72 h sensitised 2780CP to cisplatin by 5.5 fold (10 μg ml$^{-1}$, 35.9 μM; $P<0.01$) and 315 fold (20 mg ml$^{-1}$, 71.8 mM; $P<0.001$). However, it is clearly apparent that sensitisation is due, at least in part to the toxicity of the fatty acid itself (Figure 2).

### *Isobologram analysis*

Since the concentrations of the fatty acids used were themselves toxic the interactions were re-analysed by the isobologram method. Figure 3a shows the concentrations of cisplatin and γ-linolenic acid which when combined for 24 h produce a 50% reduction in survival of cell line 2780CP. The dotted line delineates the envelope of additivity. It is clear that the majority of points lie within the envelope indicative of an additive interaction. Figure 3b shows the concentrations of doxorubicin and eicosapentaenoic acid which when combined for 24 h produce a 50% reduction in survival of cell line 2780AD. Again the interaction appears to be additive. Similar isobolograms were obtained when cells were exposed to the fatty acids before exposure to the cytotoxic drugs.

### Discussion

These results show clearly that both γ-linolenic acid and eicosapentaenoic acid are toxic to tumour cells lines. The polyunsaturated fatty acids were shown in some cases to sensitise the cells to cytotoxic drugs. However, when the drug interactions were analysed by the isobologram method the interactions were clearly additive and not supra-additive.

Surprisingly, the doxorubicin and cisplatin resistant ovarian cell lines were cross resistant to the fatty acids although the resistance factors were much smaller than those for the selecting agent. Cross resistance was not a general feature of multidrug resistant cell lines since the doxorubicin resistant breast cell line showed a tendency towards greater sensitivity to the fatty acids than the parental cell line and this supports previous observations (Sircar *et al.*, 1990).

The results are consistent with previous studies which have shown that polyunsaturated fatty acids have direct antineoplastic activities and that tumour cells differ in their sensitivities to polyunsaturated fatty acids (Abou *et al.*, 1988; Karmali *et al.*, 1985). The mechanisms by which polyunsaturated fatty acids kill cells is unknown but there is evidence to suggest that toxicity may be related to lipid peroxidation and

**Table II** Sensitivities of the ovarian cell lines to cisplatin when exposed for 24 h in the presence or absence of polyunsaturated fatty acids

| | | $ID_{50}$(μM) A2780 | | 2780AD | 2780CP |
|---|---|---|---|---|---|
| *Cisplatin* | | 0.49 ± 0.08 | | 5.0 ± 0.3*** | 10.5 ± 0.6*** |
| + γ-linolenic acid | (2.5) | 0.36 ± 0.05 | (10) | 4.7 ± 0.3 | 12.2 ± 0.8 |
| (μg ml$^{-1}$) | (5) | 0.13 ± 0.04** | (20) | 3.0 ± 0.3** | 9.4 ± 0.5 |
| | (10) | 0.06 ± 0.01** | (40) | 0.82 ± 0.15*** | 6.1 ± 0.8* |
| + eicosapentaenoic | (2.5) | 0.46 ± 0.06 | (10) | 6.2 ± 0.5 | 8.3 ± 0.5* |
| acid (μg ml$^{-1}$) | (5) | 0.60 ± 0.05 | (20) | 3.9 ± 0.7 | 8.0 ± 0.3* |
| | (10) | 0.40 ± 0.02 | (40) | ND | 8.1 ± 1.0* |

Values are the mean ± standard error of triplicate determinations from one representative experiment. The concentration (μg ml$^{-1}$) of the fatty acid used was lower for A2780 than for the other two cell lines and is shown in parentheses. Statistically significant differences in sensitivity when compared with exposure to cisplatin alone are shown by asterisks (*$P<0.05$; **$P<0.01$; ***$P<0.001$). ND = not done.

**Table III** Sensitivities of the ovarian cell lines to cisplatin and doxorubicin when exposed to polyunsaturated fatty acids for 48 h before exposure to cisplatin or doxorubicin alone for 4 h

| | | | $ID_{50}$ (μM) | | |
|---|---|---|---|---|---|
| | | *A2780* | | *2780AD* | *2780CP* |
| *Doxorubicin* | | 0.022±0.001 | | 8.9±0.5 | 0.080±0.006 |
| + γ-linolenic acid (μg ml$^{-1}$) | (2.5) | 0.016±0.003 | (40) | 3.5±0.2** | 0.023±0.004** |
| + eicosapentaenoic acid (μg ml$^{-1}$) | (2.5) | 0.018±0.004 | (40) | 0.4±0.1*** | 0.013±0.002** |
| *Cisplatin* | | 0.79±0.01 | | 13.2±0.5 | 26.8±0.8 |
| + γ-linolenic acid (μg ml$^{-1}$) | (2.5) | 0.50±0.06* | (40) | 19.0±1.7 | 3.3±1.1*** |
| + eicosapentaenoic acid (μg ml$^{-1}$) | (2.5) | 0.65±0.14 | (40) | 8.7±1.7 | 2.7±0.6*** |

Values are the mean ± standard error of triplicate determinations from one representative experiment. The concentration (μg ml$^{-1}$) of the fatty acid used was lower for A2780 than for the other two cell lines and is shown in parentheses. Statistically significant differences in sensitivity when compared with exposure to cisplatin or doxorubicin alone are shown by asterisks (*$P<0.05$, **$P<0.01$, ***$P<0.001$).

**Figure 2** Dose response curves for cell line 2780CP exposed to cisplatin for 4 h after pretreatment of the cells with a γ-linolenic acid for 72 h. Three concentrations of γ-linolenic acid were used (shown as μg ml$^{-1}$) and cell survival is expressed as absorbance per well. Results are the mean ± standard error of triplicate plates.

**Figure 3** Isobolograms for the interaction between γ-linolenic acid and cisplatin in cell line 2780CP **a**, and eicosapentaenoic acid and doxorubicin in cell ine 2780AD **b**. Cells were exposed to the cytotoxic drug for 24 h in the presence of the fatty acid. The $ID_{50}$ was used to define a relative drug concentration of 1 and the dotted lines define the envelope of additivity. Points are the concentrations (expressed relative to the $ID_{50}$ concentration) of the two agents which when combined kill 50% of the cells.

production of superoxide radicals (Begin *et al.*, 1988; Das *et al.*, 1987). This is supported by the observation that the toxicity of γ-linolenic acid in human neuroblastoma cells *in vitro* is inhibited by antioxidants (Fujiwara *et al.*, 1984). The most cytotoxic polyunsaturated fatty acids appear to be those with 3, 4 or 5 double bonds and these include eicosapentaenoic acid and γ-linolenic acid. Docosahexaenoic acid which has six double bonds is much less toxic to cells (Begin *et al.*, 1986*a*). Furthermore, eicosapentaenoic acid and γ-linolenic acid are more potent in terms of lipid peroxidation and superoxide radical formation than docosahexaenoic acid. Part of the selective toxicity of polyunsaturated fatty acids to tumour cells might be explained by altered activities of enzymes that metabolise these acids. Both delta 6- and 5-fatty acyl-CoA desaturases have been shown to be impaired or absent in some tumour cell lines (Howards & Howard, 1974; Maeda *et al.*, 1978).

Initial studies of the interactions between polyunsaturated fatty acids and the cytotoxic drugs were designed to determine whether there 'is an acute effect of the fatty acid on cellular drug sensitivity. Co-incubation of cells with the polyunsaturated fatty acids had varying effects on cell survival depending on the cell line and the fatty acid used. The most significant interactions were observed between γ-linolenic acid and cisplatin. γ-Linolenic acid sensitised all three ovarian cell lines to cisplatin and the effect was dose dependent (Table II). This combination was, however, least effective in the cisplatin resistant cell line 2780CP. In contrast, eicosapentaenoic acid had no effect on the sensitivity of cell lines A2780 and 2780AD to cisplatin but produced a slight increase in sensitivity in cell line 2780CP (1.3 fold, $P<0.05$, Table II). Neither of the polyunsaturated fatty acids had any effect on doxorubicin sensitivity of the three cell lines.

In an attempt to enhance the sensitising effects of the polyunsaturated fatty acids cells were pre-incubated with the polyunsaturated acids before exposure to the cytotoxic drugs. The rational behind this approach was based on the assumption that pretreatment with the fatty acids would have one of two effects. Either they would make the cells more sensitive to subsequent exposure to a second cytotoxic agent or pre-exposure would allow the fatty acids to become incorporated into the cellular lipids and thus alter membrane fluidity. Exposure to the polyunsaturated fatty acids for 24 h before exposure to cisplatin was less effective for 2780AD than co-exposure. However, it should be noted that for these experiments the exposure time to cisplatin and doxorubicin was reduced to 4 h and this may well explain this difference.

A 48 h pre-treatment period had marked effects on the sensitivity of 2780CP to cisplatin (Table III). Both eicosapentaenoic acid and γ-linolenic acid were equally effective (10 and 8 fold sensitisation respectively $P < 0.001$). This was surprising since γ-linolenic acid was more effective than eicosapentaenoic acid when a 24 h pretreatment period was used (5.6 fold c.f. 1.4 fold). Both polyunsaturated fatty acids sensitised 2780AD and 2780CP to doxorubicin when cells were pre-treated for 48 h ($P < 0.01$, Table III).

In order to ensure that the polyunsaturated fatty acids would be incorporated into cellular phospholiopds at 72 h pre-treatment protocol used by Zijlstra *et al.* (1987) and Timmer-Bosscha *et al.* (1989) was followed. For these experiments the concentration of the fatty acids was reduced and the fatty acid was replaced after 48 h. The stability of eicosapentaenoic acid and γ-linolenic acid in culture meidum is not known so the exact exposure time may be less than 72 h. Only the interactions between eicosapentaenoic acid and doxorubicin in 2780AD and between γ-linolenic acid and cisplatin in 2780CP were examined and the results were consistent with the trends already observed for the shorter pre-exposure periods. However, these experiments also show clearly that part, if not all, of the apparent sensitisation is due to the toxicity of the fatty acid alone (Figure 2). Thus at the highest concentration of γ-linolenic acid used for pre-treatment of 2780CP the fatty acid alone killed more than 50% of the cells and it was not possible to determine an $ID_{50}$ concentration for cisplatin. If cell survival is expressed as a percentage of the control where the control for γ-linolenic acid exposure is cells exposed to γ-linolenic acid alone for 72, it is possible to compare treatments. However, the parameter derived from this approach is not an $ID_{50}$. It is assumed that the effect of γ-linolenic acid alone on cell survival is constant regardless of the concentration of cisplatin used. Clearly when studying the interaction of two drugs it would be wrong to assume that γ-linolenic acid increases cisplatin toxicity but not *vice versa.*

Since the toxicity of the fatty acid alone was apparent in all pretreatment experiments drug interactions were subject to a more critical analysis. The isobologram method described by Steel and Peckham (1979) was originally designed for studies of the interactions between radiation and cytotoxic drugs (Steel, 1979) and it has been used successfully to study interactions between a variety of agents (Carter & Wampler, 1986; Church *et al.*, 1988; Gessner, 1988). Application of this method of analysis to the interaction between γ-linolenic acid and cisplatin showed clearly that the two agents demonstrate additive toxicities (Figure 3a) since the majority of the points lie within the envelope of additivity. Additive toxicities were also demonstrated for eicosapentaenoic acid and doxorubicin (Figure 3b). These observations are entirely consistent with previous reports that polyunsaturated fatty acids can sensitise drug resistant cell lines to cytotoxic drugs (Timmer-Bosscha *et al.*, 1989; Zijlstra *et al.*, 1987). Zijlstra *et al.* (1987) showed that the doxorubicin resistant cell ine $GLC_4$/ADR was more sensitive to docosahexaenoic acid than the parental cell line $GLC_4$ and was sensitised to doxorubicin by 72 h pretreatment with the fatty acid whereas $GLC_4$ was not sensitised. Similarly, docosahexaenoic acid sensitised the cisplatin resistant subline $GLC_4$ CP to cisplatin, but did not sensitise $GLC_4$ (Timmer-Bosscha *et al.*, 1989). The authors were unable to explain this sensitisation since cellular platinum concentrations, total DNA bound platinum and the amount of the major DNA-platinum adducts increased in both cell lines. The sensitisation could well be explained if $GLC_4CP$, like $GLC_4$/ADR, is more sensitive to docosahexaenoic acid than $GLC_4$.

These results suggest that eicosapentaenoic acid and γ-linolenic acid do not have a role as resistance modulators in drug resistant cell lines. However, additive toxicity can be of great value in the clinic provided that the toxicities of the two drugs in normal tissues are non-additive. High concentrations of polyunsaturated fatty acids have been given to both animals and man without apparent toxicity (Dodge, 1990; Van der Merwe *et al.*, 1987; Zhu *et al.*, 1989). Since it is known that tumour cells derive most of their fatty acids from the host circulation it should be possible to deliver polyunsaturated fatty acids to tumour cells, perhaps preferentially. This aspect has already been exploited using eicosapentaenoic acid in an attempt to reduce the cachexia associated with growth of some tumours and, indeed, inhibition of tumour growth has been observed (Beck *et al.*, 1991; Tisdale & Beck, 1991).

### References

ABOU, E.-E.S.H., PRASSE, K.W., CARROLL, R., WADE, A.E., DHARWADKAR, S. & BUNCE, O.R. (1988). Eicosanoid synthesis in 7, 12-dimethyl-benz(a)-anthracene-induced mammary carcinomas in Sprague-Dawley rats fed primrose oil, menhaden oil, or corn oil diets. *Lipids*, **23**, 948–954.

ANDREWS, P.A. & HOWELL, S.B. (1990). Cellular pharmacology of cisplatin: perspectives an mechanisms of acquired resistance. *Cancer Cells*, **2**, 35–43.

BASU, A., KOZIKOWSKI, A.P., SATO, K. & LAZO, J.S. (1991). Cellular sensitisation to cis-diamminedichloroplatinum (II) by novel analogues of the protein kinase C activator lyngbyatoxin A. *Cancer Res.*, **51**, 2511–2514.

BECK, S.A., SMITH, K.I. & TISDALE, M.J. (1991). Anticachectic and antitumour effect of eicosapentaenoic acid and its effect on protein turnover. *Cancer Res.*, **51**, 6089–6093.

BEGIN, M.E., ELLS, G., DAS, U.N. & HORROBIN, D.F. (1986*a*). Differential killing of human carcinoma cells supplemented with n-3 and n-6 polyunsaturated fatty acids. *J. Natl Cancer Inst.*, **77**, 1053–1062.

BEGIN, M.E., DAS, U.N. & ELLS, G. (1986*b*). Cytotoxic effects of essential fatty acids in mixed culture of normal and malignant human cells. *Prog. Lipid Res.*, **25**, 573–576.

BEGIN, M.E., ELLS, G. & HORROBIN, D.F. (1988). Polyunsaturated fatty acids-induced cytotoxicity against tumour cells and its relationship to lipid peroxidation. *J. Natl Cancer Inst.*, **80**, 188–194.

CARTER, W.H. & WAMPLER, G.L. (1986). Review of the application of response surface methodology in the combination therapy of cancer. *Cancer Treat. Rep.*, **70**, 133–140.

CHURCH, M.W., DINTCHEFF, B.A. & GESSNER, P.K. (1988). The interactive effects of alcohol and cocaine on maternal and foetal toxicity in the Long-Evans rat. *Neurotoxicol. Teratol.*, **10**, 355–361.

DAS, U.N., HUANG, Y.S., BEGIN, M.E., ELLS, G. & HORROBIN, D.F. (1987). Uptake and distribution of cis-unsaturated fatty acids and their effect on free radical generation in normal and tumour cells *in vitro. Free Radicals Biol. Med.*, **3**, 9–14.

DODGE, J.A. (1990). Essential fatty acids in cystic fibrosis. In *Omega-6 Essential Fatty Acid: Pathophysiology and Roles in Clinical Medicine*. Horrobin, D.F. (ed.), pp. 427–435. Alan-Liss: New York.

FINE, R.L., PATEL, J. & CHABNER, B.A. (1988). Phorbol esters induce multidrug resistance in human breast cancer cells. *Proc. Natl Acad. Sci. USA*, **85**, 582–586.

FUJIWARA, F., TODO, S. & IMASHUKU, S. (1984). Antitumour effect of gamma-linolenic acid on cultured human neuroblastoma cells. *Prostaglandins Leukot. Med.*, **15**, 15–34.

GESSNER, P.K. (1988). A straightforward method for the study of drug interactions: an isobolographic analysis primer. *J. Am. Cell. Toxicol.*, **7**, 987–1012.

HOFMANN, J., DOPPLER, W., JAKOB, A., MALY, K., POSCH, L., UBERALL, F. & GRUNICKE, H.H. (1988). Enhancement of the antiproliferative effects of cis-diamminedichloroplatinum (II) and nitrogen mustard by inhibitors of protein kinase C. *Intl. J. Cancer*, **42**, 382–388.

HOWARDS, B.V. & HOWARD, W.J. (1974). Lipid metabolism in cultured cells. *Adv. Lipid. Res.*, **12**, 52–96.

ISONISHI, S., ANDREWS, P.A. & HOWELL, S.B. (1990). Increased sensitivity to cis-diaminedichloroplatinum (II) in human ovarian carcinoma cells in response to treatment with 12-O-tetradecanoylphorbol-13-acetate. *J. Biol. Chem.*, **265**, 3623–3627.

KARMALI, R.A., MARSH, J. & FUCHS, C. (1985). Effects of dietary enrichment with gamma-linolenic acid upon growth of the R3230AC mammary adenocarcinoma. *J. Nutr. Growth Cancer*, **2**, 41–51.

MANN, S.C., ANDREWS, P.A. & HOWELL, S.B. (1988). Comparison of lipid content, surface membrane fluidity, and temperature dependence of cis-diamminedichloroplatinum (II) accumulation in sensitive and resistant human ovarian carcinoma cells. *Anticancer Res.*, **8**, 1211–1216.

MAEDA, M., DOI, O. & AKAMATSU, Y. (1978). Metabolic conversion of polyunsaturated fatty acids in mammalian cultured cells. *Biochim. Biophys. Acta.*, **530**, 153–164.

MURRO, I. (1983). Eskimo diets and disease. *Lancet*, **i**, 1139–1141.

PETERSON, R.H., MEYERS, M.B., SPENGLER, B.A. & BEIDLER, J.L. (1983). Alteration of plasma membrane glycopeptides and gangliosides of Chinese hamster cells accompanying development of resistance to daunorubicin and vincristine. *Cancer Res.*, **43**, 222–228.

PLUMB, J.A., MILROY, R. & KAYE, S.B. (1989). Effects of the pH dependence of 3-(4,5-dimethylthiazol-2-yl)-2, 5-diphenyl-tetrazolium bromide-formazan absorption on chemosensitivity determined by a novel tetrazolium-based assay. *Cancer Res.*, **49**, 4435–4440.

RAMU, A., GLAUBIGER, D., MAGRATH, I.T. & JOSHI, A. (1983). Plasma membrane lipid structural order in doxorubicin-sensitive and resistant P366 cells. *Cancer Res.*, **43**, 5533–5537.

RINTOUL, D.A. & CENTER, M.S. (1984). Involvement of plasma membrane lipid structural order in adriamycin resistance in Chinese hamster lung cells. *Cancer Res.*, **44**, 4978–4980.

SIEGFRIED, J.A., KENNEDY, K.A., SARTORELLI, A.C. & TRITTON, T.R. (1983). The role of membranes in the mechanism of action of the antineoplastic agent adriamycin. Spin-labelled studies with chronically hypoxic and drug-resistant tumour cells. *J. Biol. Chem.*, **258**, 339–343.

SIEGFRIED, J.M., BURKE, T.G. & TRITTON, T.R. (1985). Cellular transport of anthracyclines by passive diffusion. Implications for drug resistance. *Biochem. Pharmacol.*, **34**, 593–598.

SIRCAR, S., CAI, F., BEGIN, M.E. & WEBER, J.M. (1990). Transformation renders MDR cells more sensitive to polyunsaturated fatty acids. *Anticancer Res.*, **10**, 1783–1786.

SPECTOR, A.A. & BURNS, C.P. (1987). Biological and therapeutic potential of membrane lipid modifications in tumours. *Cancer Res.*, **47**, 4529–4537.

STEEL, G.G. (1979). Terminology in the description of drug-radiation interactions. *Intl. J. Radiation Oncology Biol. Phys.*, **5**, 1145–1150.

STEEL, G.G. & PECKHAM, M.J. (1979). Exploitable mechanisms in combined radiotherapy-chemotherapy: The concept of additivity. *Int. J. Radiat. Oncol. Biol. Phys.*, **5**, 85–91.

TIMMER-BOSSCHA, H., HOSPERS, G.A.P., MEIJER, C., MULDER, N.H., MUSKIET, F.A.J., MARTINI, L.A., UGES, D.R.A. & DE VRIES, E.G.E. (1989). Influence of docosahexaenoic acid on cisplatin resistance in a human small cell lung carcinoma cell line. *J. Natl Cancer Inst.*, **81**, 1069–1075.

TISDALE, M.J. & BECK, S.A. (1991). Inhibition of tumour-induced lipolysis *in vitro* and cachexia and tumour growth *in vivo* by eicosapentaenoic acid. *Biochem. Pharmacol.*, **41**, 103–107.

VAN DER MERWE, C.F., BOOYENS, J. & KATZEFF, I.E. (1987). Oral gamma-linolenic acid in 21 patients with untreatable malignancy. *Br. J. Clin. Pract.*, **41**, 907–915.

WHEELER, C., RADER, R. & KESSEL, D. (1982). Membrane alterations associated with progressive adriamycin resistance. *Biochem. Pharmacol.*, **31**, 2691–2693.

WRIGHT, S. & BURTON, J.L. (1982). Oral evening primrose seed oil improves atopic eczema. *Lancet*, **ii**, 1120–1122.

ZHU, Y.-P., SU, Z.-W. & LI, C.-H. (1989). Growth-inhibition effects of oleic acid, linolenic acid, and their methyl esters on transplanted tumours in mice. *J. Natl Cancer Inst.*, **81**, 1302–1306.

ZIJLSTRA, J.G., DE VRIES, E.G.E., MUSKIET, F.A.J., MARTINI, L.A., TIMMER-BOSSCHA, H. & MULDER, N.H. (1987). Influence of docosahexaenoic acid *in vitro* on intracellular adriamycin concentration in lymphocytes and human adriamycin-sensitive and -resistant small cell lung cancer cell line, and on cytotoxicity in the tumour cell lines. *Int. J. Cancer*, **40**, 850–856.